# Daily Stressors and Physiological Reactivity: The Role of Perceived Stress, Stress Reactivity, Neuroticism, and Age

**DOI:** 10.1093/geroni/igaa057.1245

**Published:** 2020-12-16

**Authors:** MacKenzie Hughes, Christopher Hertzog

**Affiliations:** Georgia Institute of Technology, Atlanta, Georgia, United States

## Abstract

Exposure to stressful events is an inevitable aspect of everyday life, such as encountering work deadlines or interpersonal conflicts. The body’s physiological stress systems can become activated when exposed to stressors, resulting in increases in cortisol from the hypothalamic-pituitary-adrenal axis and/or increases in the alpha-amylase enzyme via the sympathetic-adrenal medullary system. We predicted physiological reactivity measured by salivary assay in an ecological momentary assessment (EMA) design, using The Perceived Stress Scale (PSS; Cohen, 1988), Perceived Stress Reactivity Scale (PSRS; Schulz, Jansen, & Schlotz, 2005), neuroticism, (Big Five Inventory; John, Donahue, & Kentle, 1991), and age. Adult participants (N=163) ages 20–80 years old (M=51.8 years) completed the individual differences measures and then provided seven saliva samples per day for 10 consecutive days while also completing five stress-related surveys per day via random-prompted EMA (Scott, Sliwinski, & Blanchard-Fields, 2013). Mean aggregate alpha-amylase correlated with the PSRS (r = -.20, p < .05) but not with the PSS or with neuroticism, suggesting alpha-amylase may be a more sensitive measure of subjective stress reactivity. In contrast, mean cortisol levels were not correlated with any of those measures. Age correlated with subjective stress (r = -.23, p < .05) and stress reactivity (r = -.20, p < .05) but not with hormone levels. Multilevel modeling will be used to evaluate within-person and between-person variation in stress hormones and EMA measures of daily stress in relation to the PSS, the PSRS, neuroticism, and age.

